# Future Directions for the Organizational Readiness for Knowledge Translation (OR4KT) Tool: Response to Recent Commentaries

**DOI:** 10.15171/ijhpm.2019.03

**Published:** 2019-01-23

**Authors:** Marie-Pierre Gagnon, Randa Attieh, Sandra Dunn, Gonzalo Grandes, Paola Bully, Carole A. Estabrooks, France Légaré, Geneviève Roch, Mathieu Ouimet

**Affiliations:** ^1^Faculty of Nursing, Université Laval, Quebec City, QC, Canada.; ^2^Primary Health and Social Care Research Center, Quebec City, QC, Canada.; ^3^Population Health and Optimal Health Practices Research Unit, CHU de Québec-Université Laval Research Centre, Quebec City, QC, Canada.; ^4^CRED Research Centre – École Supérieure des Affaires, Beirut, Lebanon.; ^5^CHEO Research Institute, Centre for Practice Changing Research Building, Ottawa, ON, Canada.; ^6^Primary Care Research Unit of Bizkaia – Osakidetza, Basque Health Service, Bilbao, Spain.; ^7^Faculty of Nursing and School of Public Health, University of Alberta, Edmonton, AB, Canada.; ^8^Department of Family and Emergency Medicine, Université Laval, Quebec City, QC, Canada.; ^9^Department of Political Sciences, Université Laval, Quebec City, QC, Canada.

We are grateful to Nuño-Solinís^1^ and Puchalski Ritchie and Straus^2^ for their commentaries on our article that presents the organizational readiness for knowledge translation (OR4KT), an instrument measuring healthcare organizations’ readiness to implement evidence-informed knowledge across a variety of services.^3^ They provide useful feedback on the tool, and share ideas that contribute to advance knowledge on how to improve the implementation of evidence-informed practices in healthcare organizations by considering organizational readiness (OR) as a precursor of successful change.

Implementing evidence-informed interventions in healthcare requires organizations to be ready to initiate and support change. However, translating scientific knowledge to the ‘real-life’ care context faces several challenges. Our article describes the process for the development, transcultural adaptation, and initial content and face validation of the OR4KT instrument. The tool was initially developed based on a conceptual mapping of the dimensions and concepts proposed in previous theories and models of OR. Subsequently, a systematic review of OR measurement instruments, a Delphi exercise, and consultation with experts provided an initial pool of items. The OR4KT was then translated and tested in three contexts – Basque region of Spain, Ontario and Québec (Canada).

The final OR4KT instrument, developed and validated in English, French, and Spanish, comprises 59 items, grouped in 6 dimensions (organizational climate, context, change content, leadership, organizational support, and motivation). It can be used to gauge the potential for successful implementation of evidence-informed practices in healthcare organizations, but also to monitor progress over the course of change.^4^

Both commentaries highlight the solid theoretical background and the involvement of stakeholders in the design of the OR4KT as major strengths of this tool. Indeed, we acknowledge previous theoretical and empirical work that made possible the development of the OR4KT. In that sense, as Puchalski Ritchie and Straus point out^2^, the dimensions of motivation, leadership, change content, and organizational climate for change measured by the OR4KT correspond to the concepts of change valence and change efficacy found in Weiner’s theory.^5^ The dimensions of organizational support and context reflect constructs found in other frameworks such as the Texas Christian University-ORC^6^ and the Promoting Action on Research Implementation in Health Services (PARiHS).^7^

As highlighted in the commentaries, other instruments have been developed in recent years to assess OR in healthcare but none has been extensively applied and validated. The OR4KT does not aim to replace other tools, and can be used in complementarity with them. Nevertheless, the OR4KT could be more easily adapted to various settings and different types of change due to its comprehensive theoretical foundations that encompass the main determinants of OR for change proposed in the literature.

However, as noted by Nuño-Solinís,^1^ the OR4KT is not intended to provide a roadmap of specific strategies that could be implemented in order to increase readiness for change in healthcare organizations, nor the success of change efforts. Other tools, such as the CFIR-ERIC Matching Tool,^8^ suggest relevant implementation strategies corresponding to implementation determinants. Thus, the OR4KT could be used as a diagnostic tool to prioritize particular implementation strategies in a given organization. Thus, mapping evidence-based implementation and change management strategies to the specific determinants of OR assessed through the dimensions and sub-dimensions of the OR4KT would greatly contribute to the field of implementation science.

Another limitation to using of the OR4KT in practice is the fact that the 59-item version could still be somewhat lengthy to be implemented in busy practices. The original version of the OR4KT had 91 items, which was considered too long by the experts and potential users consulted during the initial validation process. Thus, we proceeded to item reduction through consensus with a panel of seven experts during the initial validation of the OR4KT, resulting in the 59-item version that was deemed acceptable by users.^4^ However, confirmatory factorial analysis with a large sample could allow reducing the number of items.

The English version of OR4KT has been applied in the context of maternal and newborn care in Ontario, and its length was also acceptable for users. Fifteen experts including four researchers, eight clinicians (with medical, nursing and midwifery experience), and three analysts (with biostatistical and epidemiological expertise) participated in three review rounds to validate the tool. The majority of respondents completed the questionnaire in 15 to 20 minutes. However, the French version of the tool was validated using a vignette that described the potential implementation of an electronic personal health record in Québec, and has not been applied yet in a real-life implementation context. Thus, further validation of this version is needed in order to assess its acceptability in practice.

Since the OR4KT is not indented to be used by all staff of a healthcare organization, only managers and other organizational members with a stake in the proposed change are expected to complete the questionnaire. However, we need more empirical testing of the tool in order to identify the best way to administrate it. For instance, we could compare the response patterns between samples of different sizes and composition.

The commentaries present the OR4KT as a valuable and useful tool with good initial measurement properties, but that needs to be tested in other settings to increase its validity and generalizability. Researchers and decision-makers are welcomed to use the OR4KT – freely available in 3 languages – to study OR for implementing research-based knowledge in healthcare organizations. Given the great interest in the OR4KT from the international scientific community, its translation to other languages, notably in Portuguese, is foreseen in the coming years. We also plan to apply the OR4KT in the context of low- and middle-income countries. Finally, mapping evidence-informed implementation and change management strategies to the dimensions and sub-dimensions of the OR4KT, and proposing effective actions that could be initiated in order to increase OR for change constitute relevant future developments to improve knowledge translation in healthcare organizations.

## Acknowledgements

We want to thank Professor Jeremy Grimshaw, principal investigator of the KT Canada project, for his essential support to the realization of this work.

## Ethical issues

Not applicable.

## Competing interests

Authors declare that they have no competing interests.

## Authors’ contributions

MPG wrote the first draft of the manuscript. All authors commented and contributed to the final manuscript.

## Funding

This project is supported by a team grant operated by Knowledge Translation Canada and offered by the Canadian Institutes of Health Research (CIHR) in partnership with the Canada Foundation for Innovation (CFI) (grant # 200710CRI-179929-CRI-ADYP-112841). MPG is Tier 2 Canada Research Chair in Technologies and Practices in Health. FL is Tier 1 Canada Research Chair in Shared Decision Making and Knowledge Translation.

## Authors’ affiliations

^1^Faculty of Nursing, Université Laval, Quebec City, QC, Canada. ^2^Primary Health and Social Care Research Center, Quebec City, QC, Canada. ^3^Population Health and Optimal Health Practices Research Unit, CHU de Québec-Université Laval Research Centre, Quebec City, QC, Canada. ^4^CRED Research Centre – École Supérieure des Affaires, Beirut, Lebanon. ^5^CHEO Research Institute, Centre for Practice Changing Research Building, Ottawa, ON, Canada. ^6^Primary Care Research Unit of Bizkaia – Osakidetza, Basque Health Service, Bilbao, Spain. ^7^Faculty of Nursing and School of Public Health, University of Alberta, Edmonton, AB, Canada. ^8^Department of Family and Emergency Medicine, Université Laval, Quebec City, QC, Canada. ^9^Department of Political Sciences, Université Laval, Quebec City, QC, Canada.
